# Equilibrium and Kinetic Study of l- and d-Valine Adsorption in Supramolecular-Templated Chiral Mesoporous Materials

**DOI:** 10.3390/molecules26020338

**Published:** 2021-01-11

**Authors:** Yanan Huang, Alfonso E. Garcia-Bennett

**Affiliations:** 1 Department of Molecular Sciences, Macquarie University, Sydney, NSW 2109, Australia; Yanan.Huang@uts.edu.au; 2 Australian Research Council Centre for Nanoscale Biophotonics, Macquarie University, Sydney, NSW 2109, Australia

**Keywords:** chiral mesoporous silica, adsorption, enantiomeric separation, kinetic

## Abstract

Adsorption kinetic studies are conducted to investigate the potential to use chiral mesoporous materials nanoporous guanosine monophosphate material-1 (NGM-1) and nanoporous folic acid material-1 (NFM-1) for the enantiomeric separation of l- and d-valine. A pseudo-second-order (PSO) kinetic model is applied to test the experimental adsorption equilibrium isotherms, according to both the Langmuir and Freundlich models and the characteristic parameters for each model are determined. The calcined versions of both NGM-1 and NFM-1 fit the Langmuir model with maximum sorption capacities of 0.36 and 0.26 g/g for the preferred adsorption enantiomers, d-valine and l-valine, respectively. Experimental results and the analysis of adsorption models suggest a strong adsorbate–adsorbent interaction, and the formation of a monolayer of tightly packed amino acid on the internal mesopore surface for the preferred enantiomers.

## 1. Introduction

Chiral adsorption phenomena are relevant to many crystallisation processes and attract the attention of wide fields of research from abiogenesis food science [1,2]. The development of functional chiral porous surfaces could provide asymmetric support for non-chiral catalyst [3,4]. Supramolecular chirality expressed by the formation of helical fibres at nano-, meso-, or macroscopic length scales have attracted much attention for stereoselective recognition, in asymmetric catalysis, chiroptical effects, in drug delivery, and separation applications [5,6,7,8,9,10,11,12]. There is an increasing demand for enantiomerically pure compounds arising from both the chemical and pharmaceutical industries, and as a consequence, there is an equal demand for technologies to separate racemic mixtures [13].

A number of chiral microporous materials such as zeolites SU-32 and ITQ37 [3], metal organic frameworks (MOFs) such as POST-1, supramolecular structures based on hydrogen-bonded organic frameworks (HOFs) and covalent organic frameworks (COFs) have been developed, demonstrating various concepts of organic chiral template imprinting within inorganic and hybrid structures [3,14]. Che et al. and Tatsumi et al. have developed a range of chiral mesoporous silica (CMS) materials prepared via anionic surfactant templating routes [15,16,17]. A similar approach has also been utilised to prepare chiral mesoporous silica materials with anionic porphyrin templates and cationic organosilanes enhanced with a small amount of (*R*)-1,1′-bi-2-naphthol to achieve enantiopurity [17]. Other chiral imprinting approaches have focused on the exterior of silica fibres through the formation of soft nanocrystalline silica aggregates self-organised from linear polyethyleneimine (sPEI) supplemented with chiral tartaric acid to create a chiral mesostructured surface [18].

Despite these new materials, analytical separation platforms utilising commercially available chiral separation media rely solely on chiral derivatising reagents with tethered chiral compounds such as cyclodextrin-based chiral selectors [19,20]. *N*-benzyl-phenethylamino-β-cyclodextrin bonded on the surface of large-pore, non-chiral mesoporous materials has been used to successfully demonstrate the use of mesoporous materials as a stationary phase in high-performance liquid chromatography (HPLC) [19,21]. Whilst these tethered mesoporous materials have excellent chromatographic performance, effectively separating β-blocker pharmaceutical compounds with separation factors as high as 1.30 within 20 min, the potential to use chiral microporous and mesoporous materials in enantiomeric separation remains unfulfilled. In part, this is due to a lack of understanding of the mechanism of chiral amplification in porous solids and its ability to impart asymmetric properties to non-chiral reagents.

We have recently developed chiral mesoporous silica materials prepared in the absence of surfactants, using chiral supramolecular templates, guanosine monophosphate (to produce nanoporous guanosine monophosphate material-1 (NGM-1)) and folic acid (to produce nanoporous folic acid material-1 (NFM-1)) [22,23]. Whilst designing these materials in a wide range of mesopore sizes remains a challenge, the potential of NGM-1 and NFM-1 as enantioselective adsorbents has been demonstrated using small-molecular-weight chiral probe molecules [24,25]. NGM-1 and NFM-1 have opposite enantiomeric selectivity when adsorbing enantiomers of tartaric acid, pinene, or valine. The adsorption process is likely to involve the formation of a monolayer coverage of adsorbate on the pore surface, which may further amplify the selectivity of the porous material during the separation process. The adsorption of amino acids as well-ordered layers on surfaces and porous materials is of importance within industrial applications involving peptide synthesis in the solid phase, the production of agrochemical and pharmaceutical products, as well as for potential applications of biomedical sensors [26].

In this work, we aim to confirm this hypothesis by investigating the enantioselective adsorption dynamics of l- and d-valine using calcined (denoted with a *c*) *c*NGM-1 and *c*NFM-1 chiral mesoporous silica materials modelled using the pseudo-second-order (PSO) kinetic equations. The adsorption equilibrium is expressed using the Langmuir and Freundlich adsorption isotherms. The Langmuir model assumes monolayer adsorptions where both the maximum valine adsorption on the porous surfaces and the equilibrium constant for specific adsorption can be estimated. The Freundlich model is used for evaluating the heterogeneous binding system of valine within the mesopore surfaces.

## 2. Materials and Methods

All chemical reagents were purchased form Sigma-Aldrich and were used as received, without further purification.

### 2.1. Synthesis of NFM-1 

The synthesis of mesoporous NFM-1 employed here is similar to that reported previously [22]. Folic acid (FA, 0.23 g, 0.18 mmol) and H_2_O (35 mL, 341.4 mmol, MillQ) were combined with a magnetic stir bar in a Teflon vessel, and the mixture was left to stir (400 rpm) for 1 h at room temperature. The stirring rate was increased to 500 rpm before the addition of 3-aminopropyl triethoxysilane (APES, 0.31 g, 0.24 mmol) followed by tetraethyl orthosilicate (TEOS, 1.18 g, 1.00 mmol). Then left under stirring for 1 h with lid on and then static for overnight. After this, the hydrothermal treatment was conducted, in which the synthesis mixture was placed into an autoclave and heated at 100 °C for one day. The yellow solid product was recovered by filtration and washed with 50 mL of distilled water, then dried at room temperature under atmospheric conditions overnight. This solid was named as *a*NFM-1 to denote the as-synthesised material. In order to remove the FA template, the synthesised sample (*a*NFM-1, 0.10 g) was extracted in a mixture of ethanol (7.0 mL) and HCl (37%, 3.0 mL) under 60 °C for a period of 12 h, the remaining solid was obtained by filtration. The extracted sample (denoted as *e*NFM-1) was calcined in air at 550 °C for a period of 6 h and was denoted as *c*NFM-1. The molar ratio of the reaction mixture for the preparation of NFM-1 is FA: H_2_O: APES: TEOS = 0.18: 341.4: 0.24: 1.00, respectively.

### 2.2. Synthesis of NGM-1 

The synthesis of mesoporous NGM-1 employed here is similar to that reported previously [22]. Initially, guanosine 5′-monophosphate disodium hydrate (GMP, 0.60 g, 0.34 mmol) was dissolved in H_2_O (21 mL, 239.55 mmol, MillQ) in a Teflon vessel. KCl (0.1345 g, 0.66 mmol) was added, and the mixture was stirred (300 rpm) for 1 h. The stirring rate was increased to 500 rpm before the addition of 3-aminopropyl triethoxysilane (APES, 0.76 g, 0.67 mmol) followed by HCl (37%, 0.28 g, 0.66 mmol). After one minute, tetraethyl orthosilicate (TEOS, 1.18 g, 1.00 mmol) was added and left under stirring for 12 h with the lid on. After this, the hydrothermal treatment was conducted, in which the synthesis mixture was placed into an autoclave and heated at 100 °C for four days. After filtration, the solid was obtained and named as *a*NGM-1 to denote the as-synthesised NGM-1. The hydrothermal treatment sample (*a*NGM-1, 0.100 g) was then extracted in a solution of ethanol (7.0 mL) and HCl (37%, 3.0 mL) under 60 °C for a period of 12 h, the remaining solid was obtained by filtration. The extracted sample (denoted as *e*NGM-1) was calcined in air at 450 °C for a period of 3 h and was denoted as *c*NGM-1. The overall molar composition of the reaction mixture for the preparation of NGM-1 is GMP: H_2_O: HCl: KCl: APES: TEOS = 0.34: 239.55: 0.66: 0.70: 0.67: 1.00, respectively. Synthesis protocols are summarised schematically in Figure S1. 

### 2.3. Adsorption Equilibrium Measurements 

Adsorption equilibrium experiments were carried out at 25 °C by varying the concentration of l- and d-valine from 50 to 500 mM. In each experiment, an accurately weighed amount of 100 mg *c*NGM-1 or *c*NFM-1 adsorbent was added to 30 mL of valine solution. The mixture was then stirred on a thermostatic mechanical hot plate operating on a constant speed at 50 rpm, the solution was filtered; after filtration, the filtrate was obtained and dried at room temperature overnight. Each filter solution was centrifuged at 8000 rpm for 5 min to remove any adsorbent particles and then circular dichroism measurement was carried out. The dried filtrates were then degassed for nitrogen adsorption–desorption isotherm and Thermogravimetric analysis (TGA) measurements. The amount of valine adsorbed at equilibrium, *q_e_*, was calculated from the mass balance equation given by

(1) $$q_{e}=\frac{V\left(C_{0}-C_{e}\right)}{m}$$

where $C_{0}$ is the initial concentration of valine in liquid phase (mg/L), $C_{e}$ is the equilibrium concentration of valine in liquid phase (mg/L), V is the volume of valine solution used (L), and m is the mass of sorbent used (g). A blank experiment was conducted with no valine under the same experiment conditions as control.

The target concentration of l- and d-valine were prepared follow the method mentioned in adsorption equilibrium measurements. Next, 100 mg of silica was added into each targeted concentration of valine solution for 24 h of continuous stirring, then this was stopped and followed by filtration and the absorbed silica was obtained by drying filter samples at room temperature overnight, collecting all the filtrate and testing by circular dichroism spectroscopy (CD). Based on the CD calibration curves, we can calculate the concentration of silica adsorbed for each isomer. Adsorption experiments were repeated 4 times and an average of the integrated area under the curve (AUC) between 190 and 234 nm was obtained at each concentration.

### 2.4. Freundlich Isotherm 

The Freundlich isotherm is the earliest known adsorption model [27]. The model applies to adsorption within pores with interactions between adsorbed molecules and adsorbent assuming an exponential decrease in adsorption energy on depletion of the adsorption centres at the surface. This isotherm is an empirical equation employed to describe heterogeneous systems and is expressed as [28,29]:

(2) $$q_{e=K_{F}C_{e}{}^{1/n_{F}}}$$

where $q_{e}$ is the equilibrium (valine) concentration on the adsorbent (g/g), $C_{e}$ is the equilibrium (valine) concentration in solution (mg/L), K_F_ is the Freundlich constant (g/L) related to the bonding energy, and 1/*n*_F_ is the heterogeneity factor. The latter is a measure of the deviation from linearity of the adsorption process, i.e., between the solution concentration and adsorption. If the value of *n*_F_ = 1, the adsorption is linear; if the value < 1, the adsorption process is chemically driven (with a strong adsorbate–adsorbent interaction or chemisorption); if the value >1, adsorption is best described as physical adsorption. A linear form of the Freundlich expression can be obtained by taking logarithms of Equation (3):

(3) $$\ln q_{e}=\ln K_{F}+\frac{1}{n_{F}}\ln C_{e}$$

Therefore, the plot of ln $q_{e}$ versus $\ln C_{e}$ generates the intercept value of $K_{F}$ and the slope 1/*n*_F_ [30].

### 2.5. Langmuir Isotherm 

The Langmuir adsorption isotherm is most widely used for a wide range of adsorbents in liquids solutions. The model includes some basic assumptions [28,29,31]: (i) the adsorption take place at specific homogenous sites within the adsorbent; (ii) one (valine) molecule occupies a single adsorption site; (iii) the adsorbent has a finite capacity for the adsorbate, where at equilibrium, a saturation point is reached where no further adsorption can occur; (iv) all sites are identical and energetically equivalent (the adsorbent is structurally homogeneous).

The equation of Langmuir is represented as follows:

(4) $$q_{e}=\frac{x}{m}=\frac{K_{L}C_{e}}{1+a_{L}C_{e}}$$

where x is the amount of valine adsorbed (mg), m is the amount of adsorbent used (g), $C_{e}$ (mg/L) and $q_{e}$ (g/g) are the liquid phase concentration and solid phase concentration of adsorbate at equilibrium, respectively, $K_{L}$ (L/g) and $a_{L}$ (L/mg) are the Langmuir isotherm constants, which are evaluated through linearisation of Equation (5):

(5) $$\frac{C_{e}}{q_{e}}=\frac{1}{K_{L}}+\frac{a_{L}}{K_{L}}C_{e}$$

hence by plotting $C_{e}/q_{e}$ against $C_{e}$ it is possible to obtain the value of $K_{L}$ from the 1/$K_{L}$ intercept and the value of $a_{L}$ from the slope, which is $a_{L}$/$K_{L}$. Graphically, a plateau characterises the Langmuir isotherm. The theoretical monolayer capacity is *q_e_* (the equilibrium adsorption of the adsorbent) and is numerically equal to $K_{L}$/$a_{L}$.

The essential features of the Langmuir isotherm can be expressed in terms of a dimensionless constant called separation factor ($R_{L}$, is the equilibrium parameter), which is defined by the following equation [32,33]:

(6) $$R_{L}=\frac{1}{1+a_{L}C_{0}}$$

where $C_{0}$ is the initial concentration (mg/L) and $a_{L}$ is the Langmuir constant related to the energy of adsorption (L/mg). The value of $R_{L}$ indicates the shape of the isotherms to be either unfavourable ($R_{L}$ > 1), linear ($R_{L}$ = 1), favourable (0 < $R_{L}$ < 1), or irreversible ($R_{L}$ = 0) [34].

### 2.6. Circular Dichroism Spectroscopy (CD) 

Circular dichroism measurements were carried out with a Jasco J-810-150S spectrometer (230 V 50/60 Hz and 270 W) using a cylindrical quartz cell (1 mm) at room temperature. Calibration curves were obtained at 204 nm using d- and l-valine as standards and using the peak areas at a range of 10 concentrations of l- or d-valine. For l-valine, the intensity varies linearly over the concentration range measured. In this method, the dependent variables are the peak area of targeted concentrations (X), while the independent variables are the enantiomers’ concentration (l- and d-valine, Y). The samples with known elemental concentrations are used to create a model relating Y to X that is used to predict the concentrations of unknown specimens. The multiple linear regression (MLR), Y = XB + E, where B is a matrix relating with the variables in such a way that minimises the square sum of the error E was used. CD measurements are included in Supplementary Materials, Figure S2.

### 2.7. Low-Angle Powder XRD 

Calcined NFM-1 and NGM-1 samples with two-dimensional hexagonal mesostructures were confirmed by powder X-ray diffractometer (D8-Discover diffractometer, Bruker, Germany) using CuKα radiation (λ = 1.5406 Å). The diffraction patterns were recorded between 1.5–60° in 2*θ* on calcined and valine-containing samples.

### 2.8. Nitrogen Sorption Isotherms

Textural properties were characterised by nitrogen adsorption–desorption isotherms using a Micromeritics TriStar II volumetric adsorption analyser (Micromeritics Instrument Corporation, Atlanta, GA, USA) measured at −196 °C. Before the measurements, samples were dried and degassed for 12 h at 100 °C for calcined samples. Specific surface areas of material were calculated by applying the Brunauer–Emmett–Teller (BET) method in the relative pressure range between 0.05 and 0.2. The total pore volume was calculated from the amount of gas adsorption at *P*/*P*_0_ = 0.95.

### 2.9. Thermogravimetric Analysis

Thermogravimetric analysis was used to determine the amount of valine adsorbed on the pore surface after adsorption experiments, using a TGA-2050 (TA instruments, New Castle, DE, USA). The analysis was done by using a temperature ramp between 20 and 900 °C at a heating rate of 20 °C min^−1^. The sample weights were 10 mg. The derivative weight loss calculation was perform using TA instruments software (TA instruments, Universal analysis 2000, version 3.0 G).

## 3. Results and Discussion

Material synthesis protocols and characterisation for *c*NGM-1 and *c*NFM-1 have been published previously [22] and are based on the use of supramolecular templates in coordination with a co-structure directing agent [35]. Figure S1 (Supplementary Materials) shows powder XRD patterns for both calcined mesoporous materials used in the present study, showing well-ordered mesostructures that can be indexed from a hexagonal unit cell. Representative scanning electron microscope (SEM, Supplementary materials, Figure S1) images show that both materials have rod-type morphologies, where *c*NFM-1 shows right-handed chiral motives (with rods >10 μm in length), whilst *c*NGM-1 shows shorter rods (≈1 μm long) and smaller diameter (≈200 nm).

**Figure 1:**
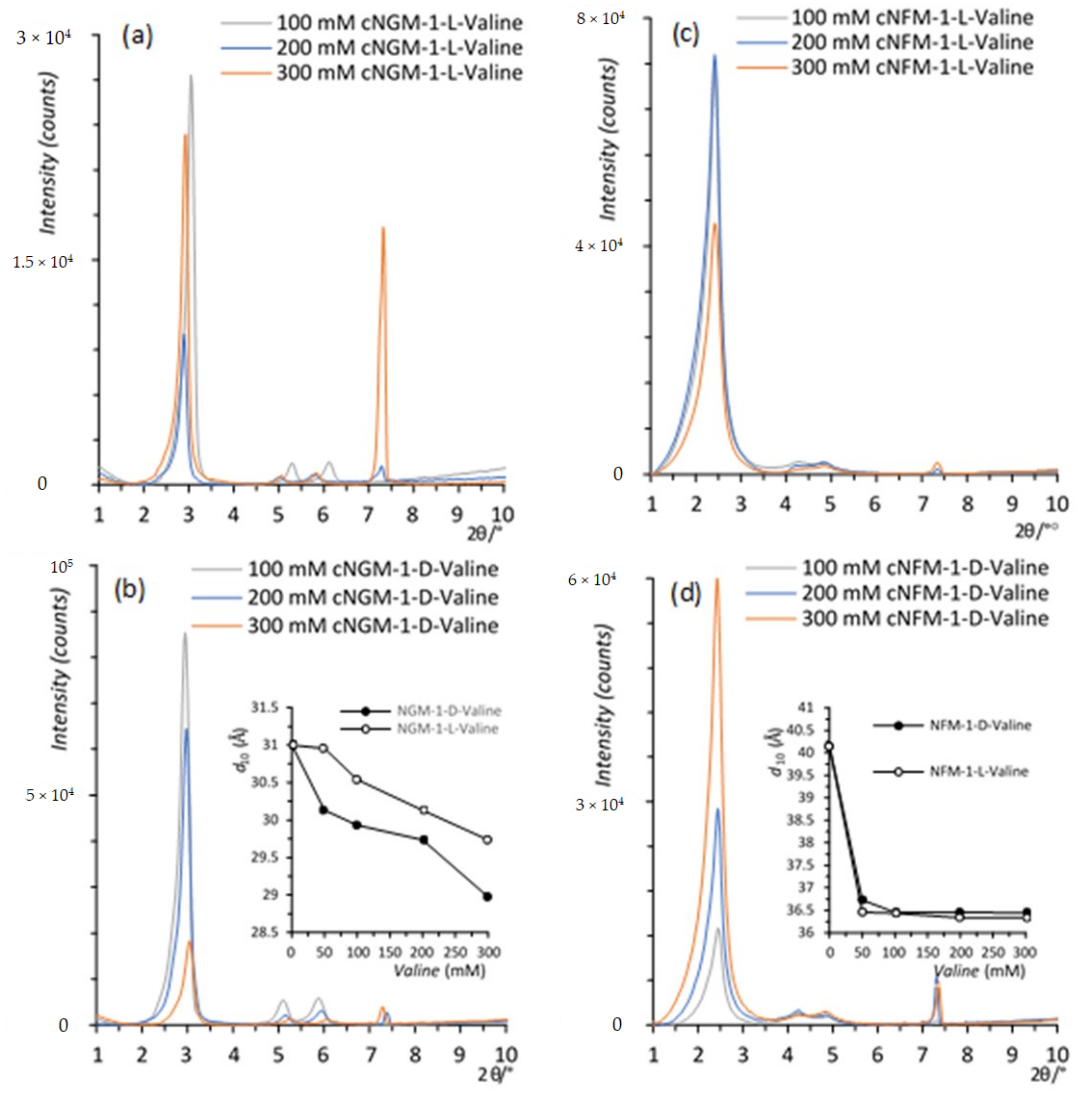
Powder X-ray diffraction patterns of mesoporous samples filtered after adsorption experiments, calcined nanoporous guanosine monophosphate material-1 (*c*NGM-1) in l-valine (**a**), and D-valine (**b**), calcined nanoporous folic acid material-1 (*c*NFM-1) in l-valine (**c**) and d-valine (**d**) at concentrations of 100, 200, and 300 mM. Inset figures in (**b**) and (**d**) show the change in the position of the (10) peak for the adsorption of each enantiomer.

Adsorption equilibrium experiments were carried out in the concentration range of 50–500 mM for l- or d-valine using a ratio of 100 mg of silica adsorbent to 30 mL of valine solution (see Section 2 and Supplementary Materials, Figure S2). After the adsorption process was completed, the mesoporous adsorbent was filtered and dried, and XRD diffractograms were recorded to confirm that there was no loss of mesoscale order. A high degree of hexagonal mesoscale order is maintained in *c*NGM-1 and *c*NFM-1 (Figure 1) evidence by mesostructured peaks corresponding to a hexagonal unit cell. There is a small decrease in the position of the (10) reflection as a function of adsorbed valine (Figure 1b,d inset), which is gradual for NGM-1, whilst remaining constant for NFM-1 samples. It is unclear why this decrease in d-spacing occurs. The formation of a homogenous coating of carbon on the internal surface of mesopores can result in changes to the scattering contrast and the formation of accidental extinctions, as observed with hexagonal mesoporous tubular hybrid carbon/silica particles, which could be contributing to the small shifts in the *d*_10_ spacing and to changes in intensity [36].

Adsorption and re-crystallisation of valine on the external surface of the silica adsorbents was assessed from the presence of a diffraction peak at ≈7.3° in 2*θ*, corresponding to the intense (002) reflection of crystalline valine [37]. The intensity of this reflection increases as a function of valine concentration. It is especially intense in *c*NGM-1(l-valine) and *c*NFM-1(d-valine) samples after adsorption of the amino acid with an initial concentration of 300 mM (see also Supplementary Materials, Figure S3). The peak is less prominent for adsorption of the opposite enantiomers at a similar starting concentration, indicating a higher adsorption capacity within the mesoporous structure and corresponding to the same enatioselectivity observed in our previous work, i.e., d-valine for *c*NGM-1 and l-valine for *c*NFM-1 [22]. Analysis of the crystallite size estimated from the Scherrer equation suggests valine crystals to be between 60 and 80 nm when the initial concentration is below 300 mM (see Table S1, Supplementary Materials).

**Figure 2:**
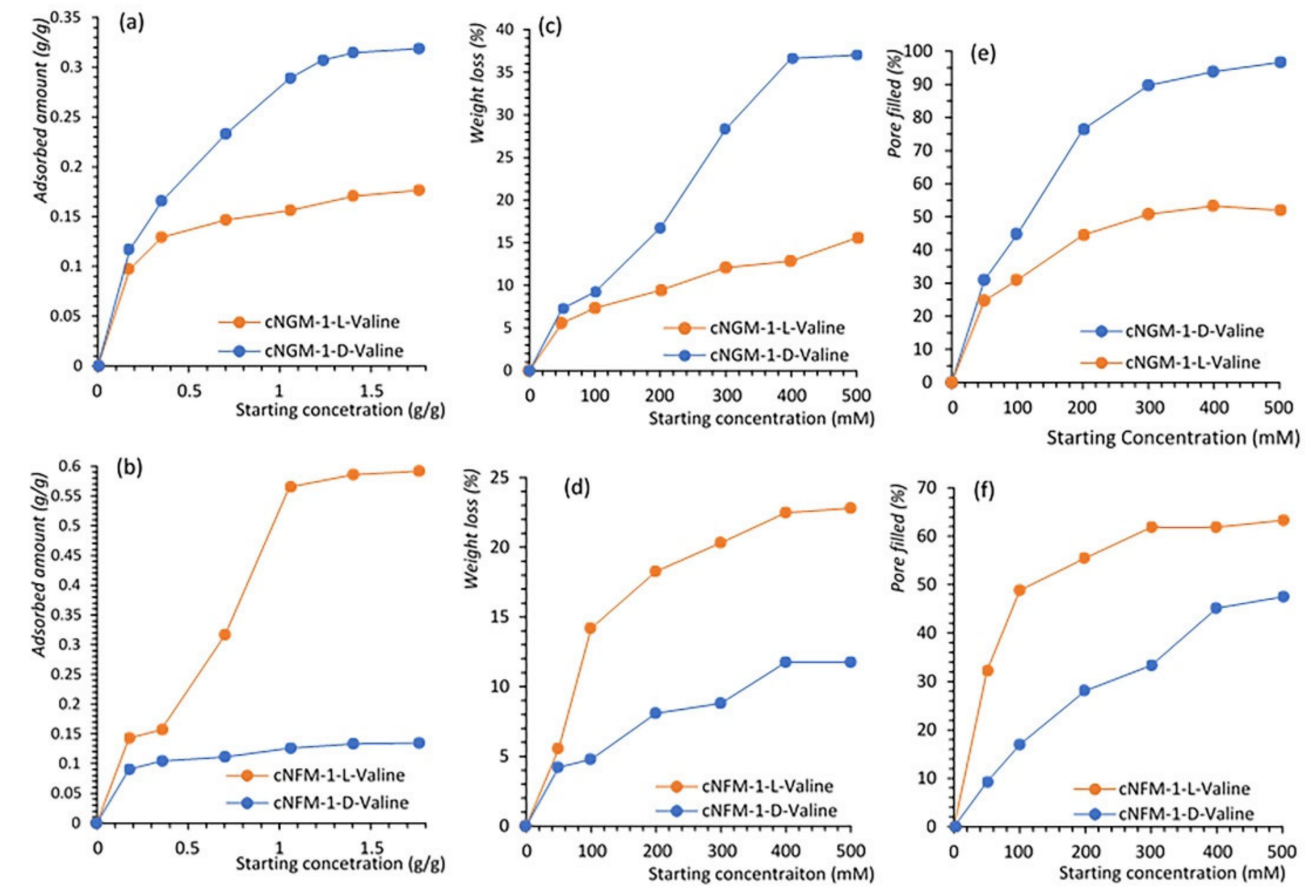
Equilibrium adsorption profiles for valine enantiomer on *c*NGM-1 and *c*NFM-1 mesoporous materials, as measured by: monitoring changes to the circular dichroism spectroscopy (CD) spectra of the valine solution after removal of the adsorbent via filtration (**a**,**b**); adsorbent percentage weight loss after filtration as measured by thermogravimetric analysis (**c**,**d**); and as a function of the change in pore volume of the filtered adsorbent as measured by nitrogen adsorption isotherms (**e**,**f**).

Figure 2a,b shows adsorption profiles for valine enantiomers on *c*NGM-1 and *c*NFM-1 quantified from changes to the CD intensity measured equilibrium at individual concentrations. For more details of this measurement, see Section 2, Materials and Methods. Profiles for all materials are characterised by a rapid onset of adsorption followed by a slower adsorption increment with increasing starting valine concentrations as the maximum adsorption is reached. The adsorption mirrors the proposed enatioselectivity, with d-valine showing a higher adsorption in *c*NGM-1 and l-valine for *c*NFM-1. The maximum adsorption capacity is 0.36 and 0.26 g/g for *c*NGM-1(d-valine) and *c*NFM-1(l-valine), respectively. These values are larger than those obtained for the basic amino acid arginine within mesoporous adsorbents (SBA-15 with 8 nm pores) and comparable to those obtained for l-histidine in mesoporous carbons [38,39]. The maximum amount of adsorbed amino acid was further confirmed from TGA conducted on the filtered mesoporous adsorbent, Figure 2c,d, with a weight loss of 28.4 wt.% for *c*NGM-1(d-valine), and 20.3 wt.% for *c*NFM-1(l-valine). Weight losses for the opposite enantiomers are lower with values of 15.7 wt.% for *c*NGM-1(l-valine) and 11.8 wt.% for *c*NFM-1(d-valine). The interaction between amino acids and the silica surface is primarily governed by electrostatic interactions. Valine’s dissociation equilibrium has pKa values at 2.32 (carboxyl group) and 9.62 (amino group) and will thus exist primarily as a zwitterion at neutral pH, as used here.

Thus, electrostatic charge matching interactions between the surface ≡SiO^−^ and R–NH_3_^+^ moiety of the amino acid can be considered as the main binding site. Additionally, –NH_3_^+^·····-COO^−^ interactions between adjacent molecules may also contribute to the adsorption process, either by promoting multilayer adsorption or limiting the diffusion of fresh adsorbate through the mesopores. Given the adsorption capacity for the preferred enantiomers and the decrease in surface as a function of adsorption, the maximum amino acid surface coverage within the pores is equivalent to 2.0 molecules per nm^2^ for *c*NGM-1 (d-valine), and 1.39 molecules per nm^2^ for *c*NFM-1 (l-valine). This could be considered a self-assembled monolayer of amino acid on the internal mesoporous surface [40].

Nitrogen adsorption isotherms (Supplementary Materials, Figure S4) conducted on the filtered and dried adsorbent were measured to assess the remaining mesoporosity within the silica adsorbents. All textural properties including surface area, pore volume, and pore width decrease, indicating the presence of the enantiomers within the mesopores. The percentage of mesopore filling was estimated by subtracting the total mesopore volume obtained for the filtered sample containing the adsorbed amino acid from that measured on the *c*NGM-1 or *c*NFM-1 (Figure 2d,e). A higher pore filling is calculated for the favoured enantiomers for each adsorbent, with approximately 96% of the pore volume filled in *c*NGM-1 (d-valine) and 63% for *c*NFM-1 (l-valine). For *c*NGM-1, such a high degree of pore filling in combination with the high surface coverage (2 molecules per nm^2^) is suggestive of amino acid packing within the mesopores.

**Figure 3:**
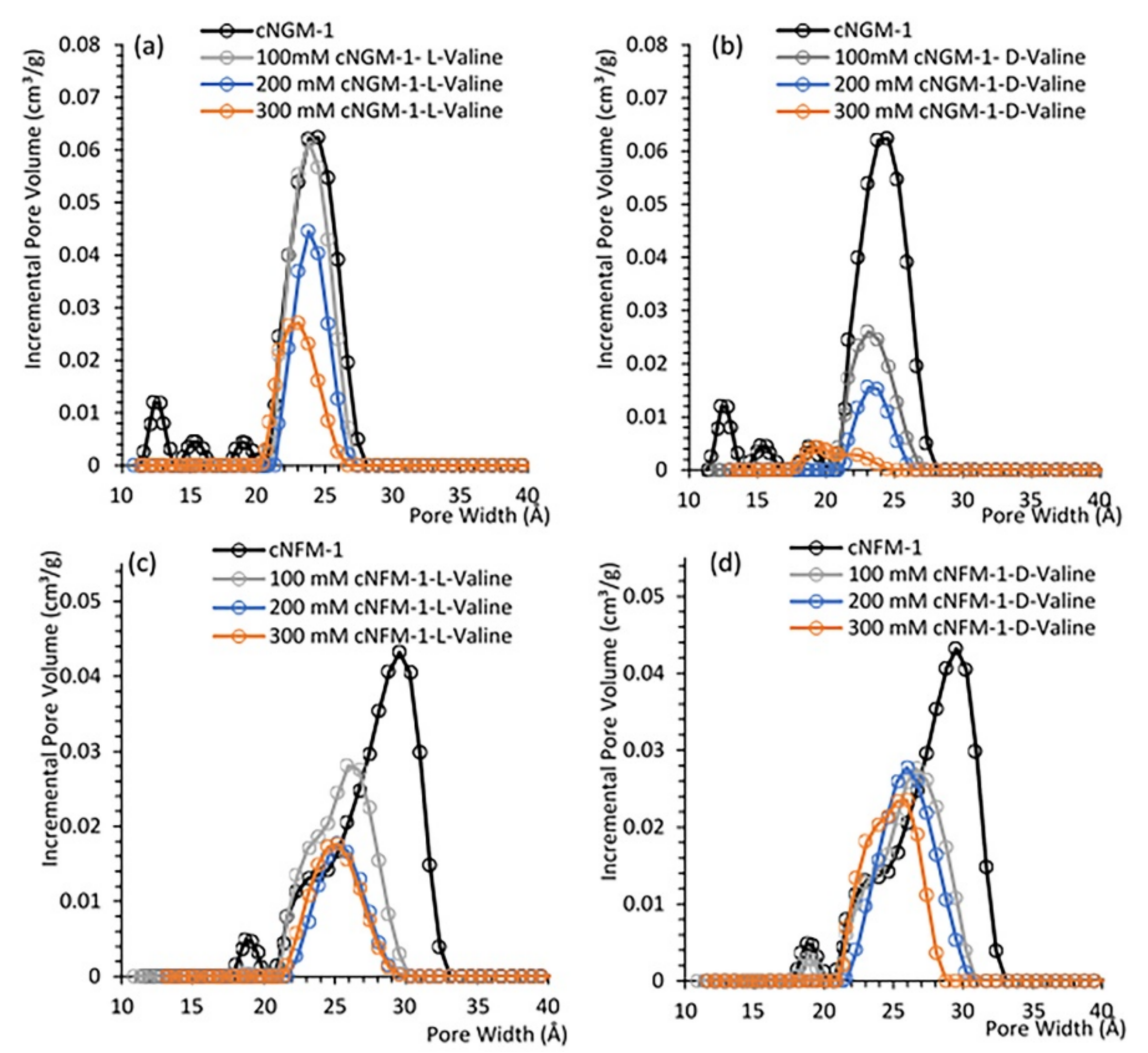
Mesopore size distribution of filtered and dried sample of: *c*NGM-1 after adsorption of (**a**) l-valine and (**b**) d-valine at concentration of 100, 200, and 300 mM; and *c*NFM-1 samples after adsorption of (**c**) l-valine and (**d**) d-valine at similar concentrations.

Significant mesopore filling of the opposite enantiomers occurs only at higher concentrations of amino acid with 50% of the mesopore volume remaining unfilled. The mesopore pore size distribution of *c*NGM-1 and *c*NFM-1 samples measured after the adsorption and filtration processes are shown in Figure 3. A decrease of <1 Å is observed for *c*NGM-1 (l-valine) even after adsorption with a starting concentration of 300 mM (Figure 3a). The mesopore size reduction for *c*NGM-1 (d-valine) after adsorption at a concentration of 300 mM is larger, ≈5 Å (Figure 3b), caused by 0.28 g/g adsorption based on TGA analysis. Similarly, adsorption of l-valine by *c*NFM-1 at a concentration of 300 mM leads to a noticeable decrease in the pore size distribution by 4.6 Å (Figure 3c), equivalent to 0.20 g/g of adsorbed amino acid in the filtered material, whilst the decrease in textural properties is less pronounced for *c*NFM-1 (d-valine) samples (Figure 3d). Adsorption at higher initial concentrations of l-valine (>400 nM) within *c*NFM-1 did not result in higher pore filling (Figure 2f) or a further reduction in mesopore size, indicating that the maximum adsorption has been reached (raw data adsorption isotherm curves are shown in the Supplementary Materials).

Adsorption data supports a mechanism based on the formation of a monolayer coverage at between 200 and 300 mM solution of l-valine and d-valine for *c*NFM1 and *c*NGM-1, respectively. Adsorption of d-valine within *c*NGM-1 at a starting solution above 400 mM leads to complete pore filling with a BET surface area of 17.5 m^2^/g remaining. In this case, multilayer coverage of the amino acid within the mesopores must be occurring. The adsorption process is different for the unfavoured enantiomers with lower adsorption capacity and a higher amount of crystalline valine (Figure 1 and Supplementary Materials, Figure S5) suggesting that a large proportion of the adsorbed unfavoured valine enantiomer resides on the external particle surface and is unable to penetrate the mesopores.

**Figure 4:**
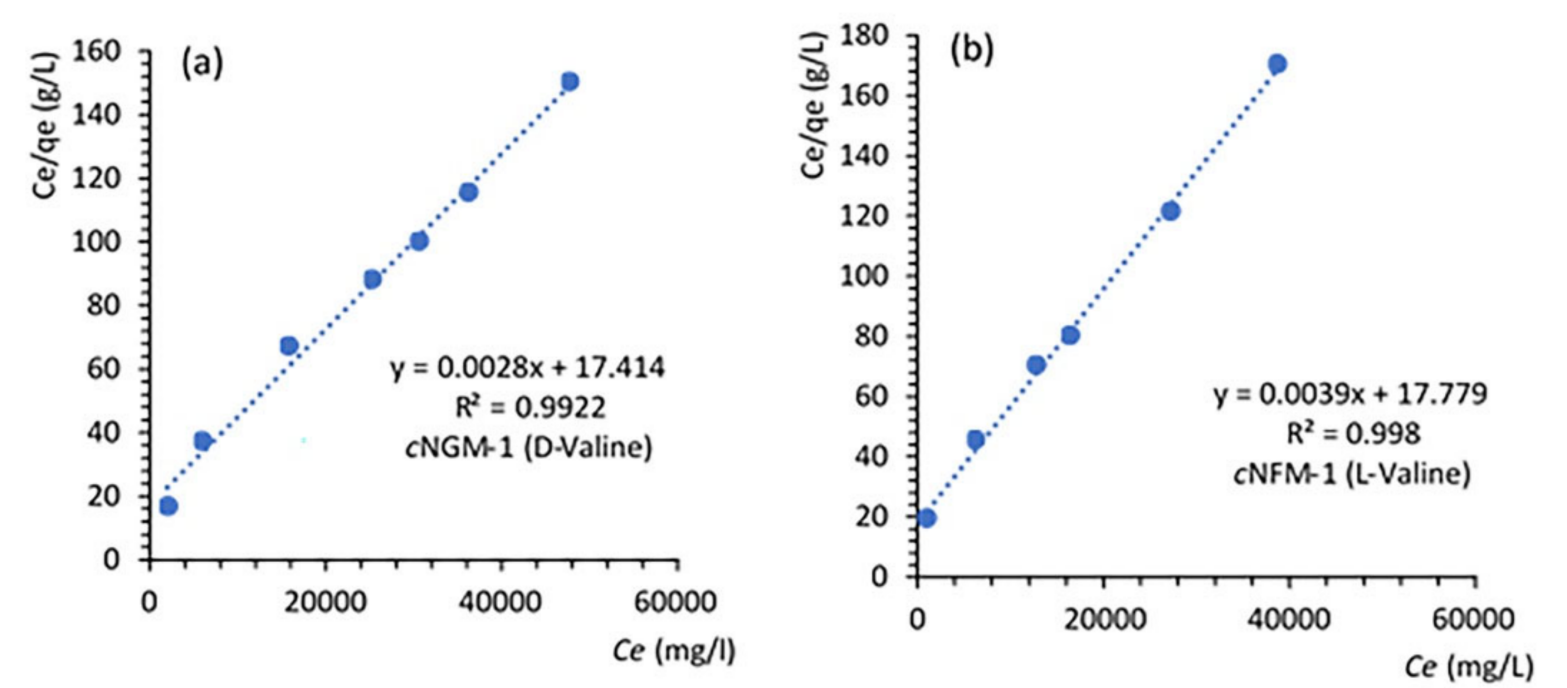
Fitting for isotherms curves based on a Langmuir isotherm model for adsorption of the favoured enantiomers of valine in (**a**) *c*NGM-1 and (**b**) *c*NFM-1 adsorbents at 25 °C.

**Figure 5:**
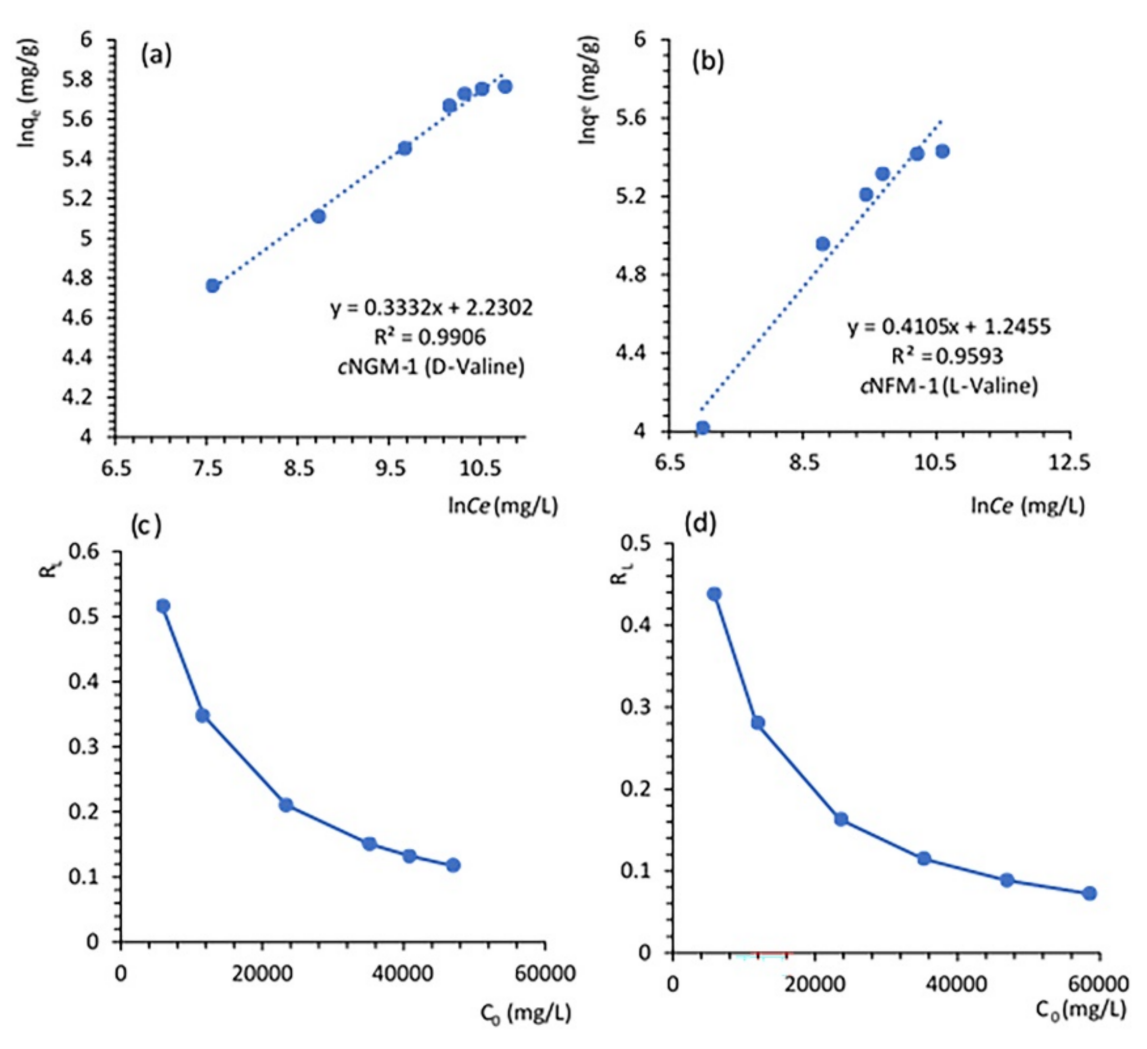
Freundlich isotherms curves and fitting data for adsorption of the favoured enantiomers of valine in (**a**) *c*NGM-1 and (**b**) *c*NFM-1 adsorbents at 25 °C. The change in separation factor as a function of valine concentration is plotted in (**c**) for *c*NGM-1 and (**d**) for *c*NFM-1.

Adsorption data were further analysed according to the linear form of the Langmuir isotherm (Equation (4) in Section 2). The plot of $C_{e}/q_{e}$ verses $C_{e}$ gives a straight line with slope $a_{L}$/$K_{L}$ and intercept 1/$K_{L}$, where $K_{L}$/$a_{L}$ gives the theoretical monolayer saturation capacity, *q_e_* (Figure 4). The calculated Langmuir constants are reported in Table 1. The isotherm curve was found to be linear over the entire concentration range studied, with a good correlation coefficient (R^2^ = 0.99 in NGM-1 and R^2^ = 0.99 in NFM-1). The monolayer saturation capacity *q_e_*, for the favoured enantiomer was determined to be 0.36 g/g for *c*NGM-1 (d-valine) and 0.26 g/g for *c*NFM-1 (l-valine). These values are comparable to the adsorption capacities determined experimentally, suggesting a homogenous distribution of active sites on the surface of the material, an important assumption of the Langmuir model. The influence of isotherm shape on a “favourable” or “unfavourable” adsorption has been considered in the literature [27]. For the Langmuir-type adsorption, the isotherm shape can be classified by a dimensionless constant separation factor (R_L_), given by Equation (5) [28]. The values of R_L_ are in the range of 0–1 (Figure 5, Table 1), confirming a favourable uptake of valine. The higher calculated R_L_ values at the lower concentration range indicate a strong surface interaction between the favoured enantiomer and the silanol surface, which rapidly decreases with increasing adsorbate concentration within the mesopores.

Linear plots for the Freundlich isotherm (Figure 5) yield a lower correlation coefficient (R^2^) value for both *c*NFM-1 (l-valine) an *c*NGM-1 (d-valine) with heterogeneity factors *n*_F_ = 2.44 and *n*_F_ = 2.96, respectively. Isotherms with n > 1 represent a high affinity between adsorbate and adsorbent [30,41]. Table 1 shows a comparison of the Langmuir and Freundlich relative parameters (K_F_, *n*_F_, and R^2^).

**Figure 6:**
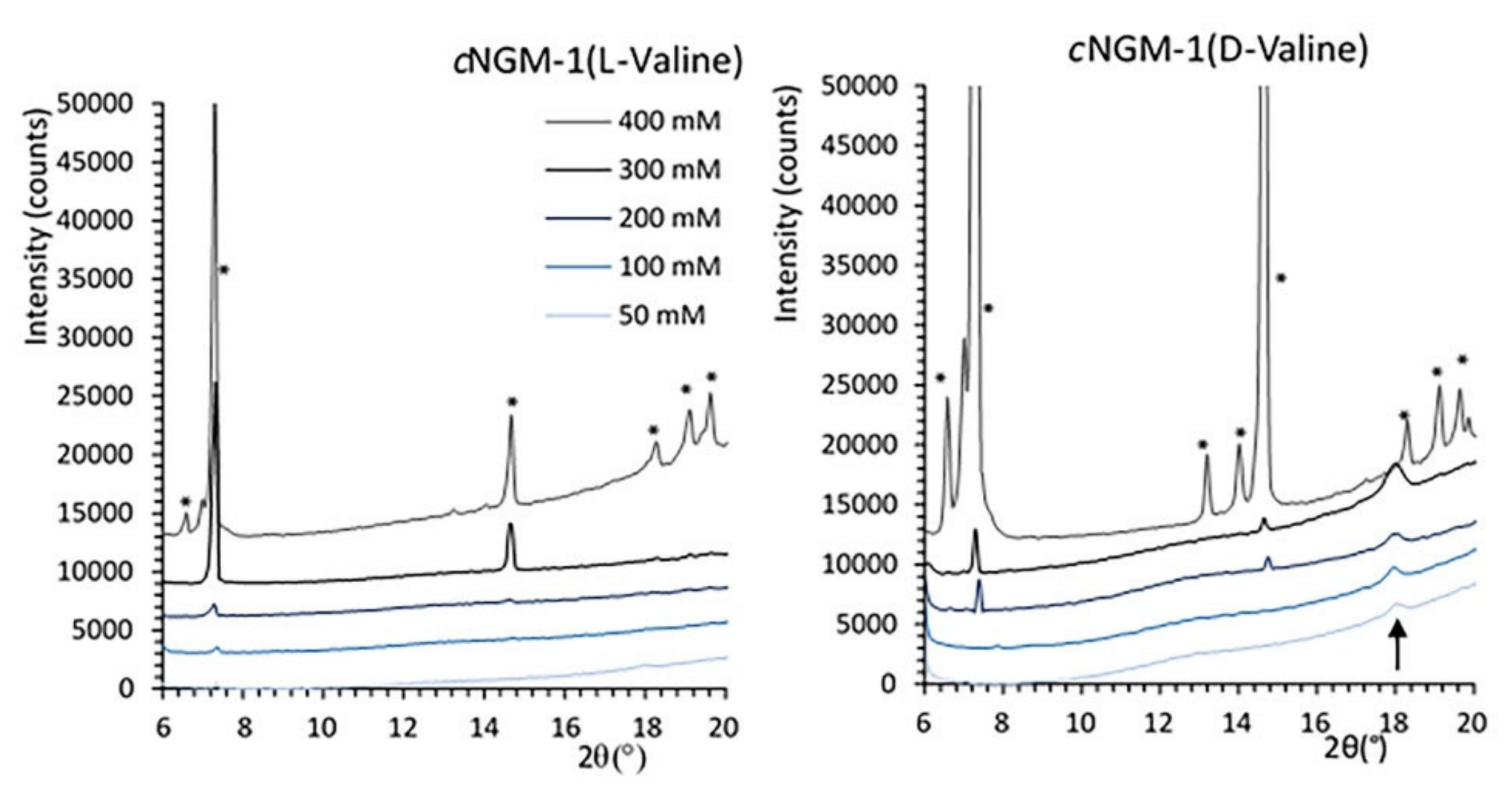
X-ray diffractograms showing the evolution of diffraction peaks associated with crystalline valine (asterisks) and a single broad peak at 18.3° (arrow) observed at lower adsorption levels of the preferred valine enantiomer only. For clarity, the diffractogram for the sample at 500 mM is not shown.

**Scheme 1:**
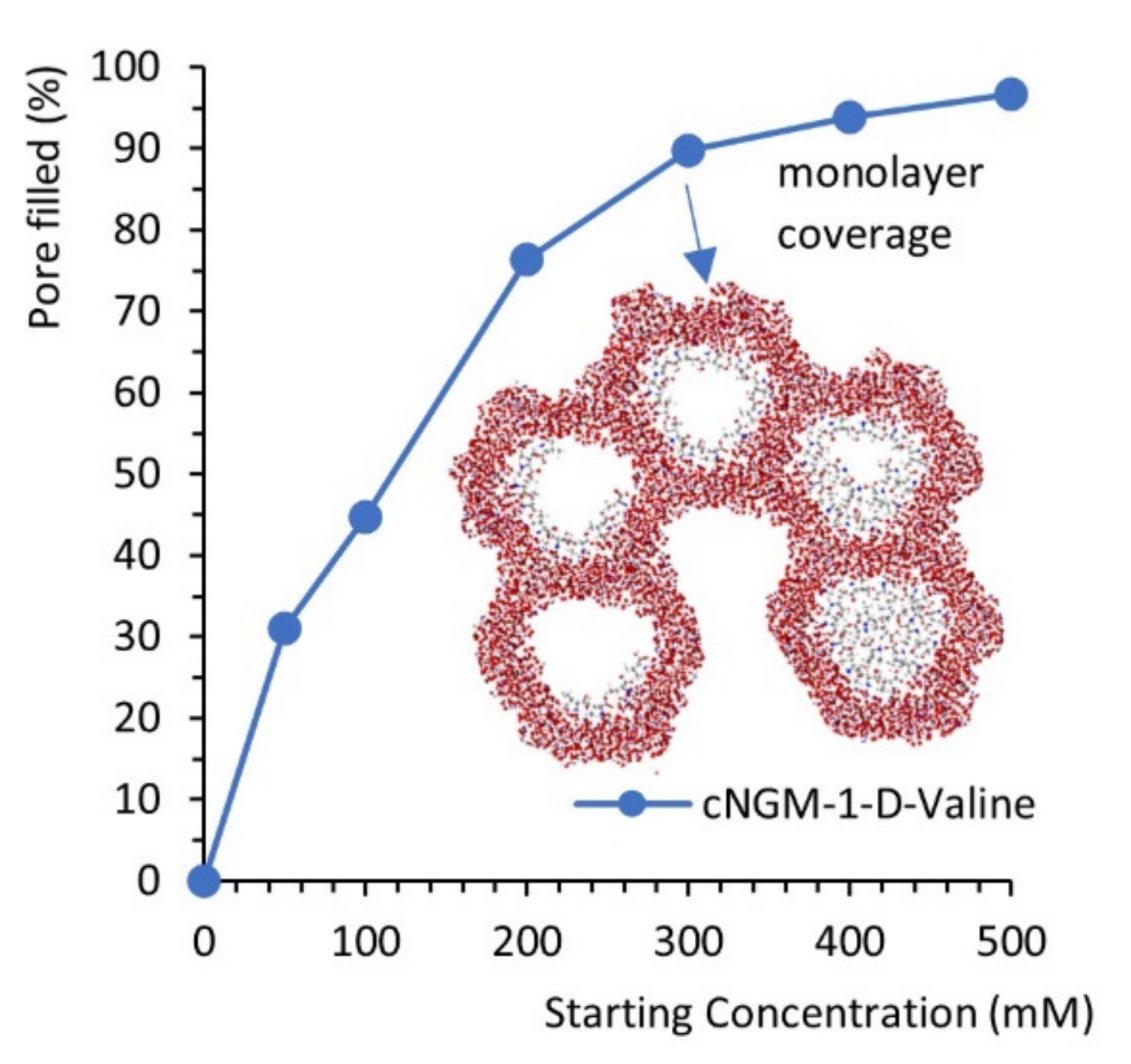
Representation of the chiral adsorption of d-Valine within the mesopores of *c*NGM-1, highlighting the different stages of adsorption process that result in a monolayer coverage of the amino acid on the surface, which retains a significant amount of porosity: and the tight packing of amino acids within the pores upon full mesopore filling.

Overall, experimental data and adsorption models indicate that *c*NGM-1 (d-valine) shows a higher adsorption capacity and a more favourable adsorption in comparison to *c*NFM-1 (l-valine), despite having a smaller pore size distribution (24.5 versus 29.5 Å). We postulate that more homogenous adsorption sites are present in *c*NGM-1 in comparison to *c*NFM-1 leading to a stronger packing of the amino acid within the mesopores. The solvent-excluded molecular volume of valine is estimated at 85.7 Å^3^ and considering the surface area and volume of a straight cylinder with height 2 Å and a radius of 12.65 Å, is ≈ 1097 Å^2^ and 943 Å^3^, respectively, the number of valine molecules per 1 nm^2^ can be approximated to 11. This is an order of magnitude higher than that measured, but considering the chiral nature of the pores and the specific supramolecular templating mechanism via a co-structure directing agent (3-aminopropyl triethoxysilane) [22,25], it is likely that some adsorption sites are sterically hindered and that a specific packing arrangement may occur within the pores. As mentioned above, there are diffraction peaks assigned to the crystallisation of the amino acid on the external surface, which are observed in the powder diffractograms (Supplementary Materials, Figure S3). The formation of crystalline material on the exterior of particles was also observed from SEM images of filtered samples (Supplementary Materials, Figure S4) at high starting amino acid concentration. Closer inspection of diffractograms of filtered samples after adsorption at lower starting concentrations (Supplementary Materials, Figure S2) reveals the presence of a prominent broad peak at 18.3° in 2*θ* observed in *c*NGM-1 (d-valine) and *c*NFM-1 (l-valine). The peak is not observed in the diffractogram of *c*NGM-1 (l-valine) samples as shown in Figure 6, and only weakly visible for *c*NFM-1 (d-valine). As this peak is not consistent with reflections from crystalline valine and appears prior to the presence of the (002) reflection at 7.3° in 2*θ*, it may be indicative of amino acid packing within the mesopores. Whilst further analysis is required to identify the origin of the diffraction peak at 18.3° in 2*θ* (*d* = 4.8 Å), it is interesting to note that the *c*NGM-1 (d-valine) samples prepared with an initial concentration of 200 and 300 mM, still retain a significant amount of accessible porosity, and its chirality may be amplified by the presence of a monolayer of tightly packed amino acid on the mesopore surface, as depicted in Scheme 1 below. Filtered materials possessing a monolayer coverage of amino acid can yield a more confined chiral environment in which other enantiomeric separations may be possible.

## 4. Conclusions

Equilibrium adsorption studies are described for the adsorption of l- or d-valine onto *c*NGM-1 and *c*NFM-1. Results confirmed the selectivity for *c*NGM-1 and *c*NFM-1 and can effectively be used as adsorbents with opposite enantioselectivity.

Experimental data indicated that the adsorption equilibrium is not dependent on the pore size of the materials. Kinetic measurements of *c*NGM-1 adsorbing in d-valine showed that the co-efficient obtained for the Langmuir model were higher than 0.99 and the values of equilibrium adsorption capacity were in better agreement with *q_e_* experimental data. The maximum adsorption capacity was 0.36 g/g at room temperature. The adsorption measurements of *c*NFM-1 with l-valine showed that the coefficient obtained also obeyed the Langmuir model. The maximum adsorption capacity was 0.26 g/g at room temperature, a little bit higher than the experimental data of 0.20 g/g. The potential chiral amplification of materials that retain some porosity is an interesting avenue to explore further as is the temperature and pH dependence of the adsorption process.

The adsorption mechanism is simultaneously dominated by surface adsorption and diffusion into the chiral pore network for the favoured enantiomers, whilst pore blockage at the pore entrance (leading to a plateau in the decrease of mesopore size reduction) occurs for the unfavoured enantiomer. These materials are limited by their pore size to smaller molecules; thus, pore size enlargement would be beneficial for the separation of larger molecules, which from a pharmaceutical perspective would be desirable.

## Figures and Tables

**Table 1:** Summary of the Freundlich and Langmuir isotherm constants and comparison of linear (R^2^) coefficients for the adsorption of the favoured enantiomers of valine in *c*NGM-1 and *c*NFM-1 adsorbents.

*c*NGM-1 (d-Valine)			*c*NFM-1 (l-Valine)	
	Parameters	R^2^	Parameters	R^2^
Freundlich	K_F_ = 8.97 L/g *n*_F_ = 2.96	0.99	K_F_ = 3.47 L/g *n*_F_ = 2.44	0.95
Langmuir	*q*_max_ = 0.36 g/g K_L_ = 0.057 L/g a_L_ = 2 × 10^−4^ L/mg R_L_ = 0.09–0.5	0.99	*q*_max_ = 0.26 g/g K_L_ = 0.055 L/g a_L_ = 2 × 10^−4^ L/mg R_L_ = 0.20–0.73	0.99

K_F_ and K_L_ and a_L_ are the Freundlich and Langmuir constants, respectively. *q*_max_ is the maximum adsorption capacity, R_L_ is the Langmuir separation factor, and *n*_F_ is the heterogeneity factor (see Section 2 for further details).

## Data Availability

Data available in a publicly accessible repository that does not issue DOIs. Publicly available datasets were analyzed in this study.

## Supplementary Materials

The following are available online, Figure S1: Synthesis protocol, powder XRD, and representative SEM images of *c*NGM-1 and *c*NFM-1, Figure S2: Calibration curves obtained from CD spectra, Figure S3: Powder XRD of valine adsorbed *c*NGM-1 and *c*NFM-1 samples, Table S1: Estimation of the crystallinity of valine from XRD, Figure S4: Nitrogen adsorption isotherms of filtered samples, and Figure S5: Representative SEM images of valine adsorbed samples.

## Sample Availability

Samples of the compounds are available from the authors.
