# Supplementary material for: Equilibrium and Kinetic Study of l- and d-Valine Adsorption in Supramolecular-Templated Chiral Mesoporous Materials
Source: Molecules. 2021 Jan 11;26(2):338. doi: 10.3390/molecules26020338 (PMC7827360; doi:10.3390/molecules26020338)
Supplement: Supplementary file 1 [file molecules-26-00338-s001.pdf]

Equilibrium and Kinetic Study of L- and D-Valine Adsorption in Supramolecular Templapted Chiral Mesoporous Materials

Yanan Huang and Alfonso E. Garcia-Bennett

Figure S1: Synthesis and characterisation of cNGM-1 and cNFM-1 **Page 1**
Figure S2: Calibration curves obtained from CD spectra **Page 2**
Figure S3: Powder XRD of Valine adsorbed cNGM-1 and cNFM-1 samples **Page 3**
Table S1: Estimation of the crystallinity of valine from XRD **Page 5**
Figure S4: Nitrogen adsorption isotherms of filtered samples **Page 6**
Figure S5: Representative SEM images of Valine adsorbed samples **Page 7**

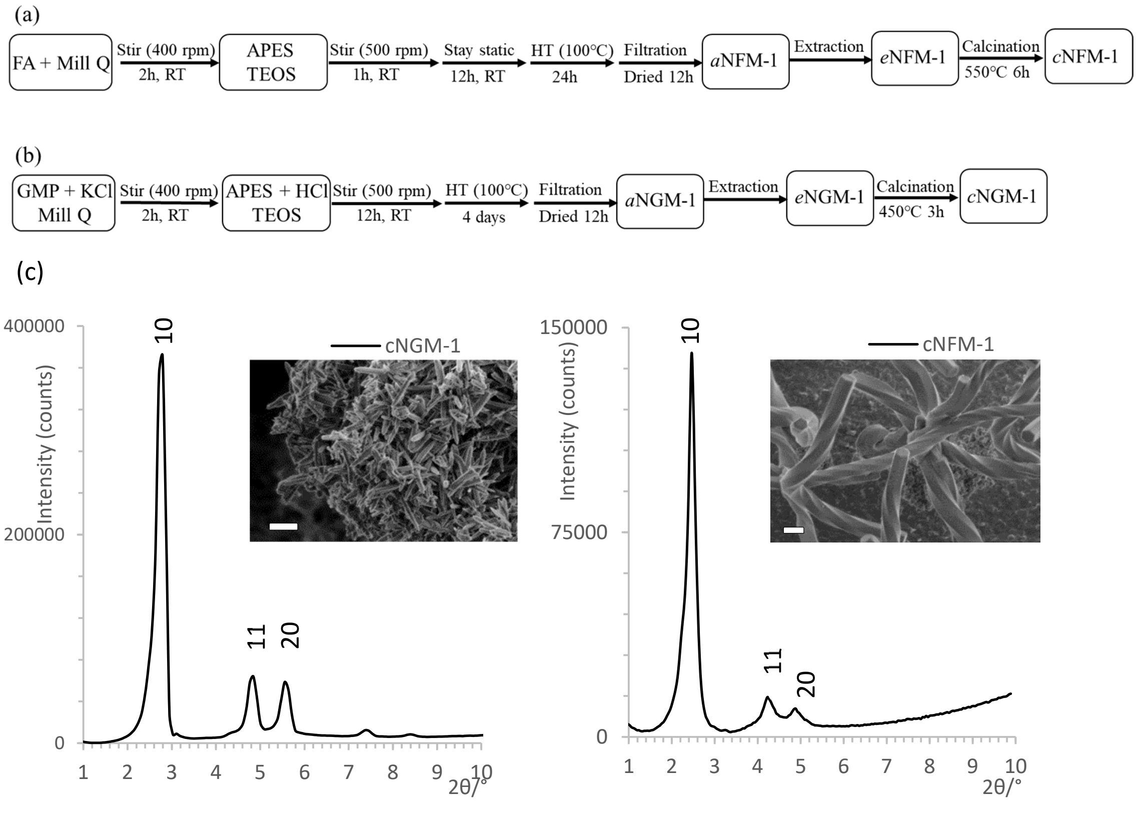

Figure S1: (a) and **(b)** Synthesis steps for the preparation of mesoporous NFM-1 and NGM-1 chiral materials from guanosine monophosphate (GMP) and folic acid (FA) supramolecular templates. TEOS=tetraethyl orthosilicate; HT=hydrothermal treatment; prefixes *a*, *e* and *c* refer to the as-synthesised, extracted, and calcined materials**. (c)** Powder X-ray diffraction (XRD) pattern of calcined NGM-1 and NFM-1 samples mesoscale diffraction peaks which can be indexed on the basis of a hexagonal $(p6mm)$ unit cell, with $a_{hex} = 2 \cdot d_{10}/\sqrt{3}$ = 36.59 Å and 41.47Å, respectively. Insets show representative SEM images for each material. Scale= 1 µm.

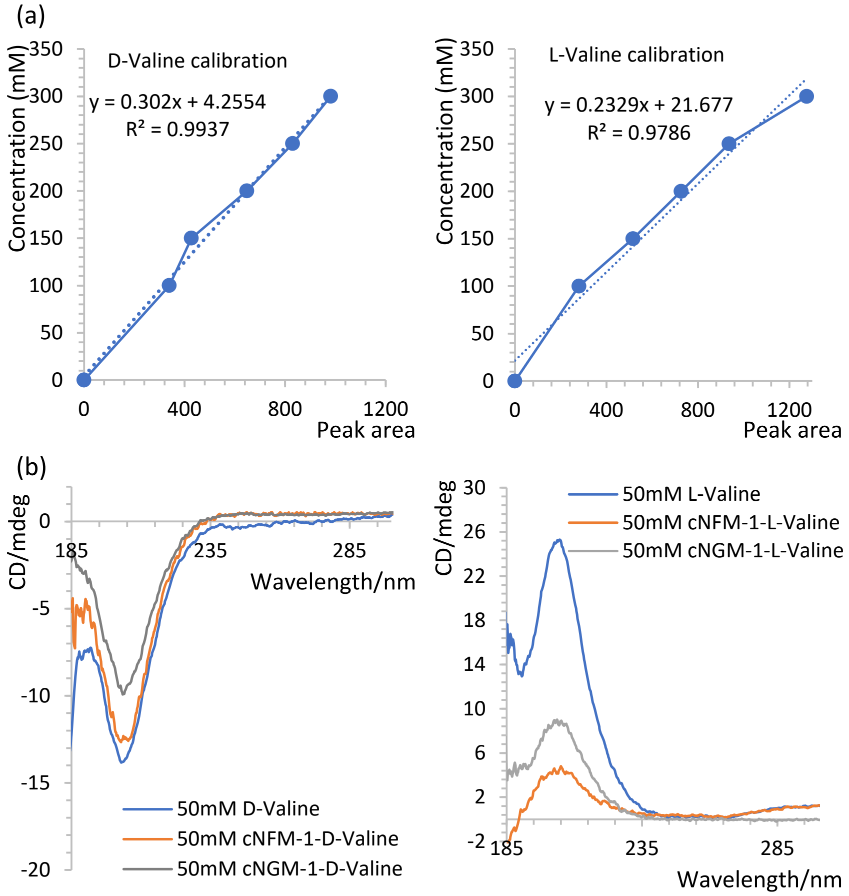

Figure S2: (a) Calibration curves obtained for D- and L-Valine standard solutions at a range of concentrations based on the intensity Circular Dichroism spectra, with a band at 204nm. The calibration curves were derived by integrating the peak areas between 190-234nm at each concentration. (b) Representative CD spectra of the valine solutions obtained from the standard amino acid, as well as from mesoporous silica filtered solutions are shown, showing the preference of adsorption for D-valine in *c*NGM-1 and L-valine for *c*NFM-1.

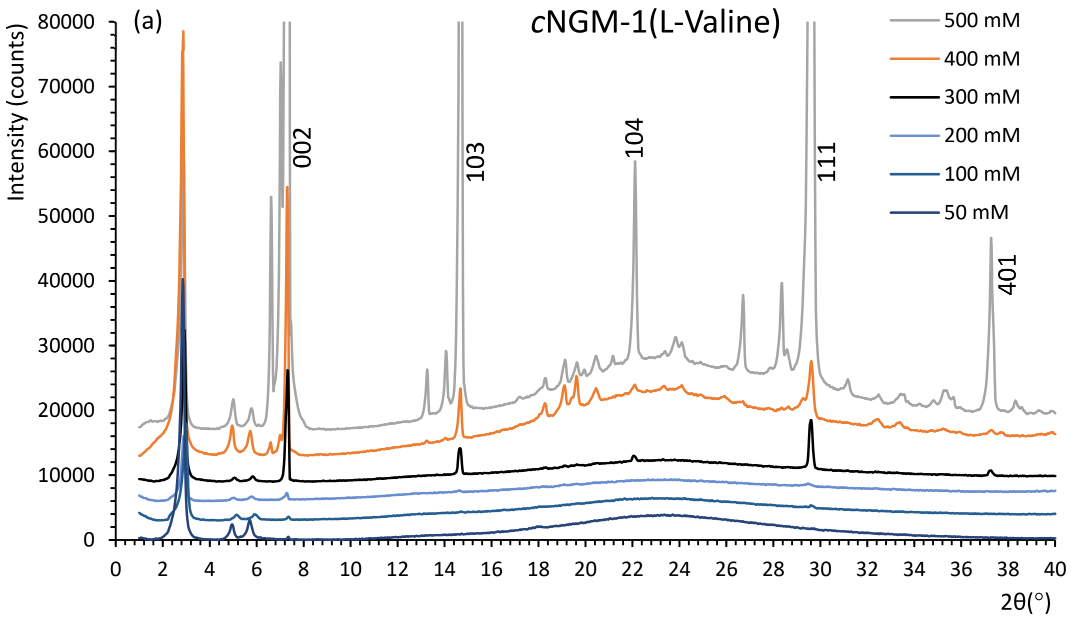

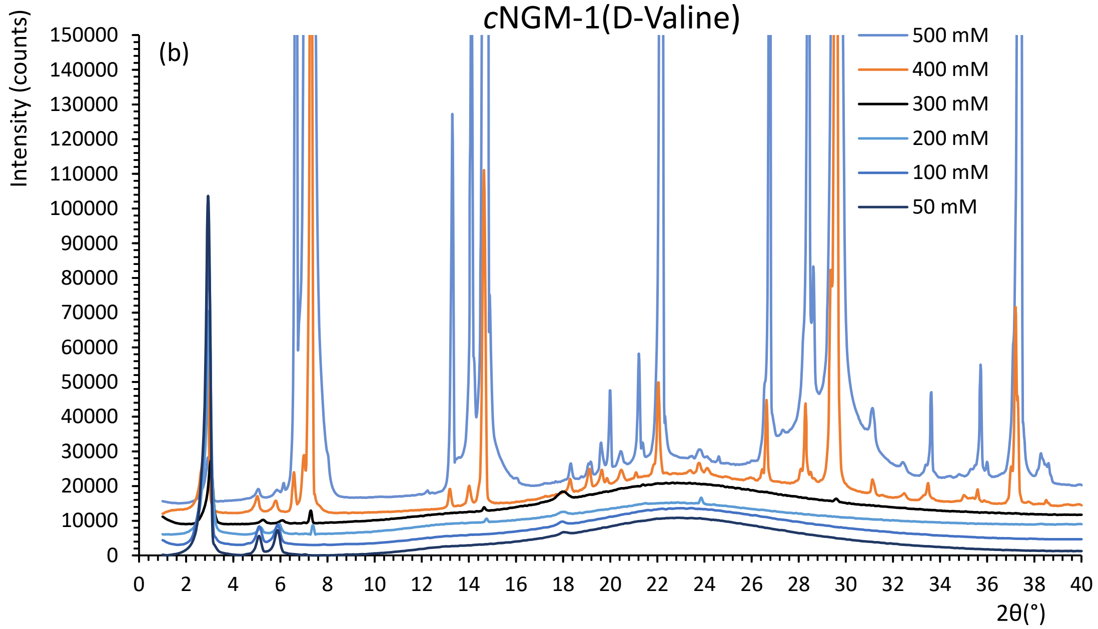

Figure S3: Powder X-ray diffraction (XRD) pattern of filtered samples of valine adsorbed within, (a) *c*NGM-1 (L-Valine), (b) *c*NGM-1 (D-Valine), (c) *c*NFM-1(L-Valine) and (d) cNFM-1 (D-Valine), at different initial concentrations of valine. Indexed high angle reflections correspond to crystalline valine (monoclinic system) on the external mesoporous particle surface. Diffractograms have been scaled by 3000 counts each, for ease of viewing.

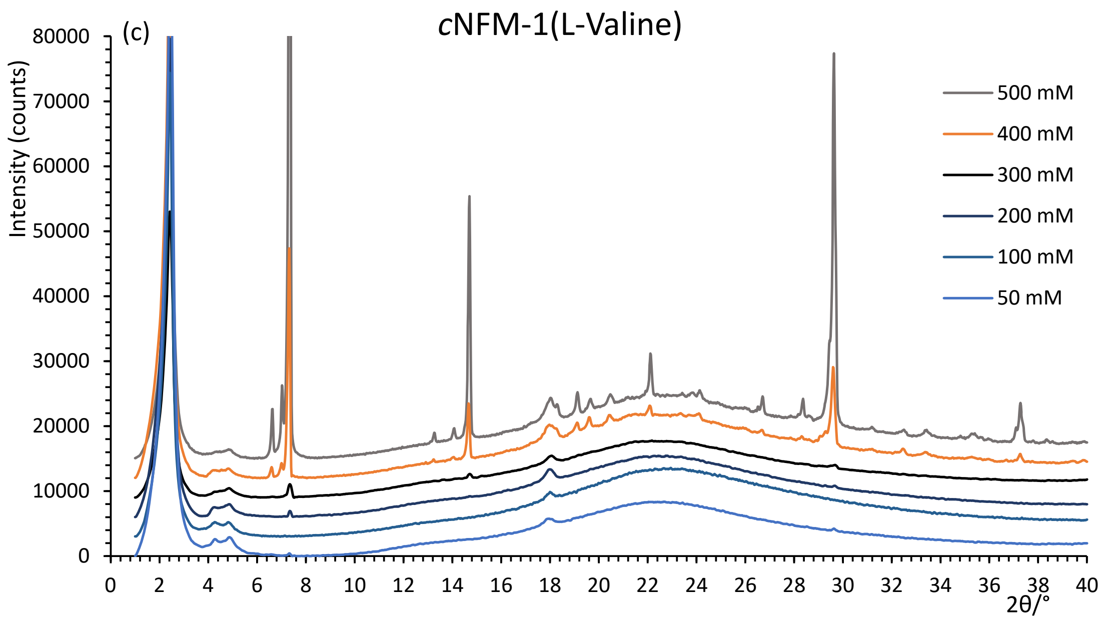
(c)
cNFM-1(L-Valine)
Intensity (counts)
80000
70000
60000
50000
40000
30000
20000
10000
0
0 2 4 6 8 10 12 14 16 18 20 22 24 26 28 30 32 34 36 38 40
2θ/°
500 mM
400 mM
300 mM
200 mM
100 mM
50 mM

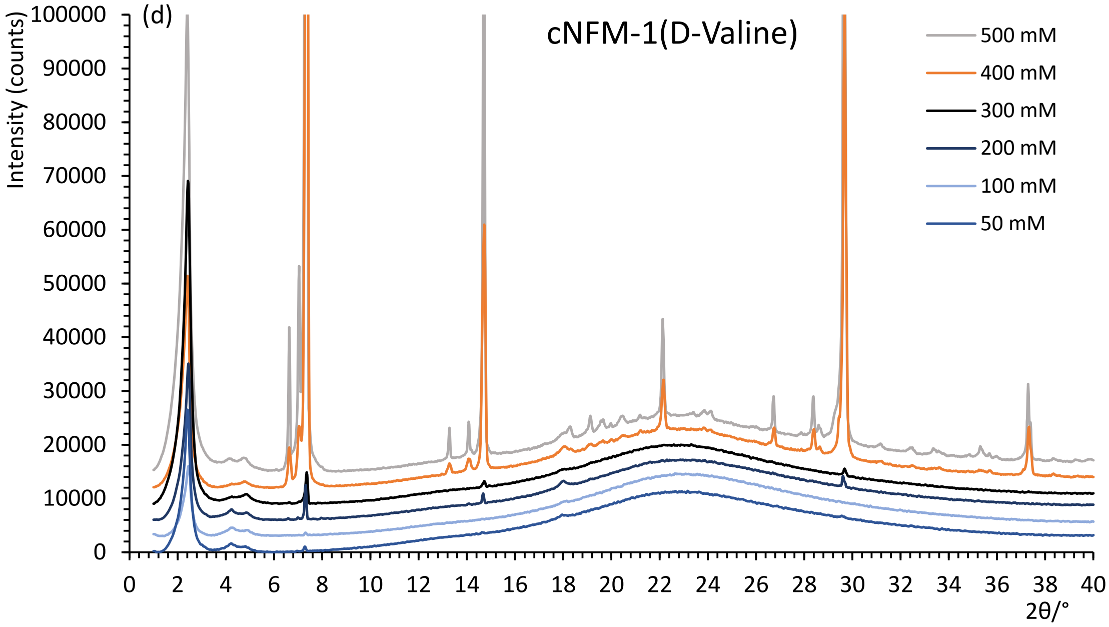
(d)
cNFM-1(D-Valine)
Intensity (counts)
100000
90000
80000
70000
60000
50000
40000
30000
20000
10000
0
0 2 4 6 8 10 12 14 16 18 20 22 24 26 28 30 32 34 36 38 40
2θ/°
500 mM
400 mM
300 mM
200 mM
100 mM
50 mM

Table S1: Estimation of the crystallinity of valine on the external particles of mesoporous materials from peak broadening using of the peak at approximately 7.3 °2θ and the Scherrer equation, Dp=Kλ/βcosθ where, Dp is the mean size of the ordered (crystalline) domains, *K* is a dimensionless shape factor (0.9 was used), λ is the X-ray wavelength, β is the line broadening at half the maximum intensity (FWHM) in radians.

Sample	Concentration (mM)	° 2θ	B, FWHM	Dp (Å)
*c*NGM-1-L-valine	100	7.3546	0.1338	62.19
	200	7.2767	0.14852	56.02
	300	7.31569	0.14785	56.28
cNGM-1-D-valine	100	7.84099	0.22504	36.99
	200	7.374	0.1184	70.28
	300	7.296	0.1199	69.40
*c*NFM-1-L-valine	100	No peak		
	200	7.335	0.1443	57.66
	300	7.335	0.15716	52.94
*c*NFM-1-D-valine	100	7.31569	0.11316	73.53
	200	7.31569	0.11523	72.21
	300	7.35468	0.10705	77.73

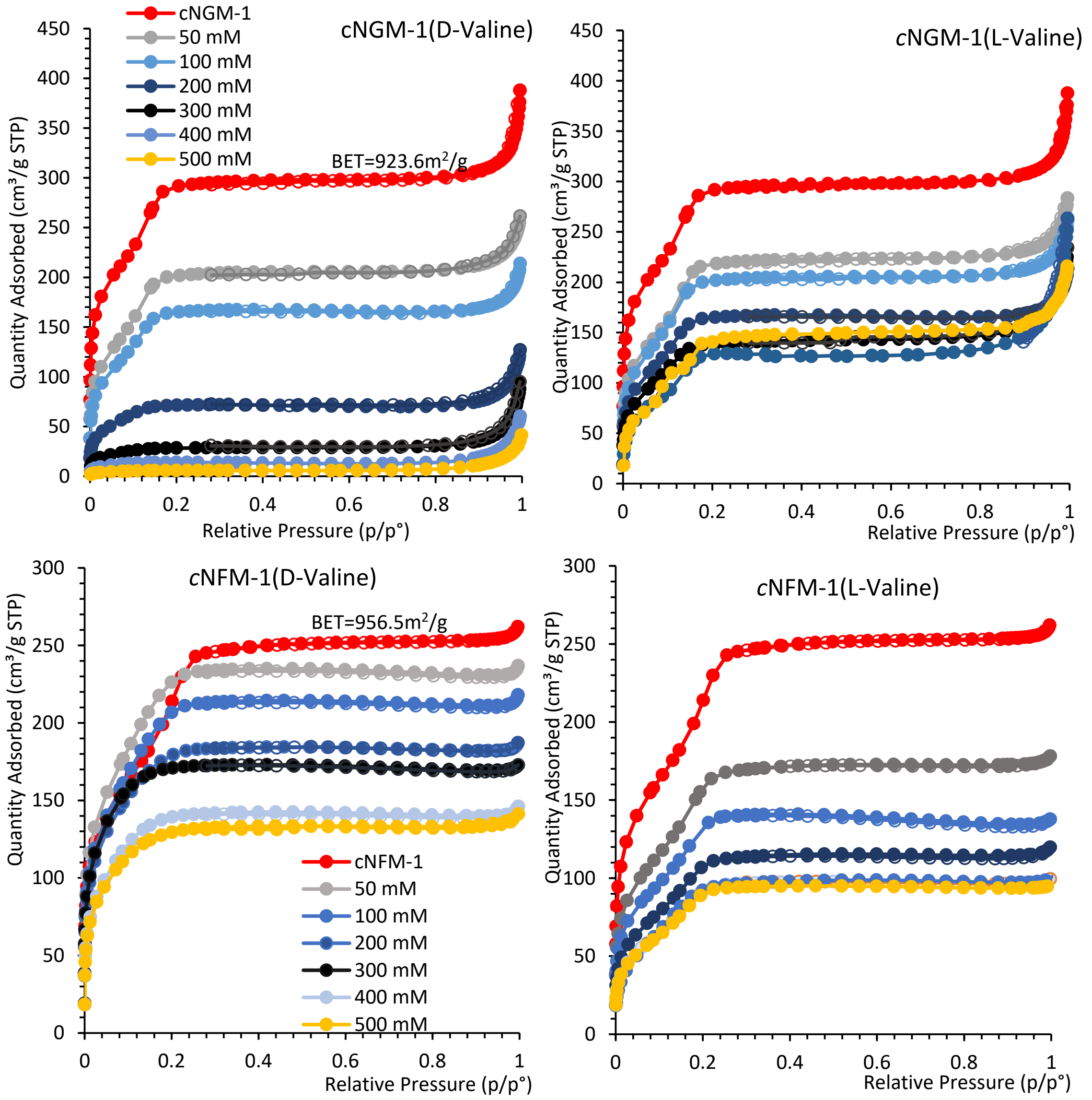

Figure S4: Nitrogen adsorption isotherms of filtered samples of valine adsorbed within mesoporous materials *c*NGM-1 and *c*NFM-1 samples at different initial valine concentrations.

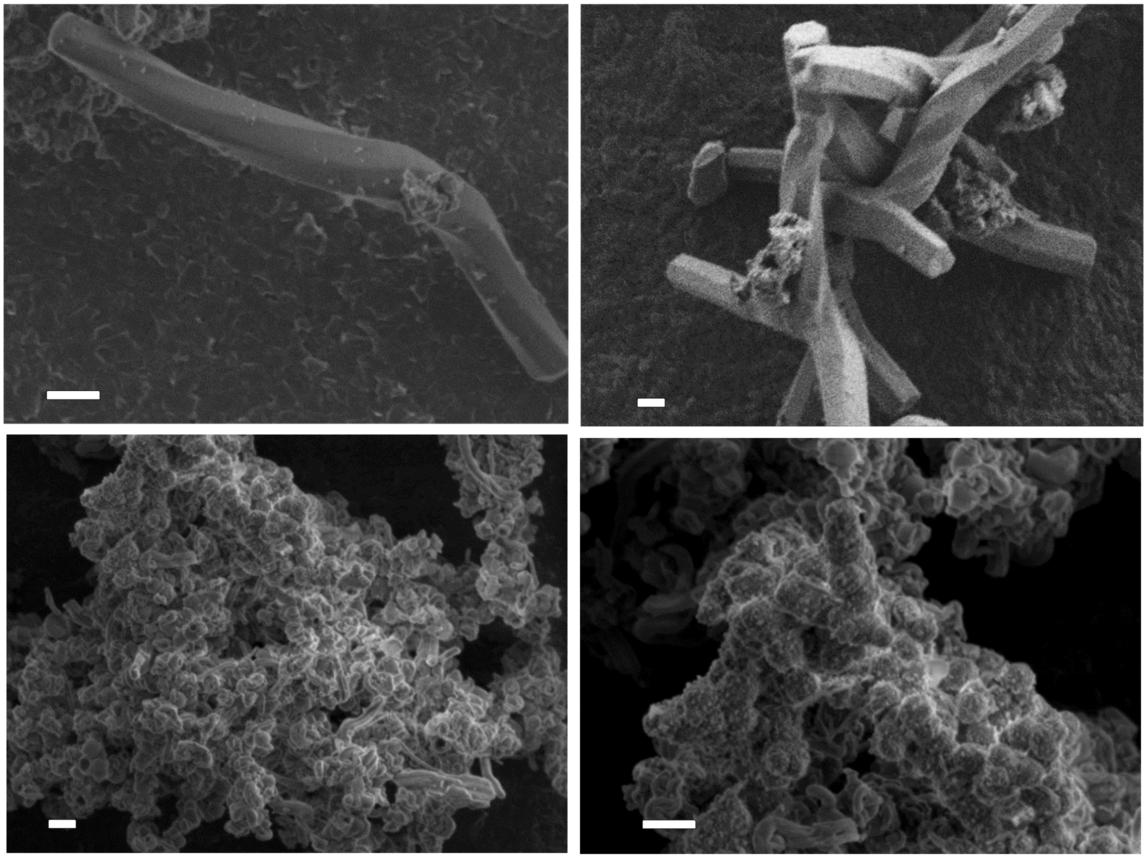

Figure S5 Representative SEM images of particles of (a,b) *c*NFM-1 (L-Valine) and (c,d) *c*NGM-1 (D-Valine) filtered and dried after adsorption of valine at an initial concentration of 300 mM. Small particles of crystalline valine can be observed on the external particle surface. Scale= 1 µm.
